# Impact of Arsenic Toxicity on Black Gram and Its Amelioration Using Phosphate

**DOI:** 10.1155/2013/340925

**Published:** 2013-07-22

**Authors:** Saumya Srivastava, Yogesh Kumar Sharma

**Affiliations:** Department of Botany, University of Lucknow, Lucknow, Uttar Pradesh 226007, India

## Abstract

The toxicity of arsenic in soil and ground water is one of the most important environmental problems particularly in South-East Asia. Arsenic-polluted irrigation water creates hazard in soil environment and also in crop quality. In the present study, response of black gram (*Vigna mungo* L.) to arsenic with or without phosphate application was investigated. Arsenic-treated plants showed reduction in their growth and pigment content. Arsenic significantly enhanced lipid peroxidation, electrolyte leakage, and level of proline showing oxidative stress. Arsenic toxicity was associated with an increase in the activities of antioxidative enzymes like superoxide dismutase, peroxidase, and ascorbate peroxidase whereas catalase activity decreased at higher arsenic dose. Joint application of phosphate with arsenic resulted in significant alterations in most of the parameters tested under the purview of arsenic treatment alone which lead to better growth in black gram.

## 1. Introduction

Arsenic (As) is a toxic metalloid [1], universally present in many environments. It is highly toxic to all forms of life. Arsenic is a group I carcinogen [2] and occurs predominantly in inorganic form as arsenate (AsV) and arsenite (AsIII). Arsenic originates from anthropogenic and geochemical sources [3]. In addition, human activities have caused an accumulation of arsenic in soils through production/use of arsenic-based pesticides [4], manufacture of arsenic based compounds, smelting of arsenic ores, mining processes, and fuel utilization [5]. Thus, human activity has exacerbated the problem of arsenic toxicity. Water supplies, soils, and sediments polluted with arsenic are the major sources of drinking water and food chain contamination in numerous countries [6, 7]. This has caused a worldwide epidemic of arsenic poisoning, with many people having developed skin lesions, cancers, and other symptoms [8, 9]. Accumulation of arsenic in human hair (180–20340 *μ*g/kg) and nails (380–44890 *μ*g/kg; [10]) in West Bengal and Bangladesh is indicative of chronic arsenic toxicity. Study shows that, besides groundwater, food is also an important pathway of arsenic in to human system [11, 12].

 Water is a very important input for crop production and if arsenic contaminated water is used for irrigation, it may create hazard both in soil environment and in crop quality. Twenty percent loss of crop (cereal) production due to high concentration (20 ppm) of arsenic in plant body was reported by Davis et al. [13]. Long-term use of arsenic laden water for irrigation result in higher arsenic levels in agricultural soils [14–16]. In this way, arsenic gets into the grains of plants, such as rice and wheat, and into vegetables and fruit plants when they are grown on arsenic contaminated soils [11, 17, 18]. The presence of arsenic in irrigation water or in soil at high levels could obstruct normal growth of plants with toxicity symptoms like biomass reduction [19], yield losses [20], inhibition of seed germination, decrease in plant height, lower fruit and grain yield [21–23], reduction in root and shoot growth [24], wilting and necrosis of leaf blades [22], and reduction in leaf area and photosynthesis [25].

 As arsenic is highly toxic metalloid and its contamination poses a serious threat to plants and animals including humans, efforts are underway worldwide to remediate arsenic contaminated soil and water. People are also working to manage arsenic toxicity in plants through various fertilization and nutrient strategies. Arsenic is analogous to phosphate (in the periodic table, both are placed in the same group, Va); both have similar electron configuration and chemical properties and compete for the same uptake carriers in the root plasmalemma [26, 27]. This suggests a possible amelioration method for arsenic toxicity. 

 In the present study, arsenic-induced toxicity in black gram was studied. We selected black gram as there appears to be very little information about its sensitivity vis-a-vis tolerance to arsenic. It is an important pulse crop in India. Black gram is a rich source of protein. It is also grown for forage and hay [28]. Its crop residues are an important feed for livestock. We studied how arsenic, at 3 concentrations: 0 *μ*M (control), 100 *μ*M, and 200 *μ*M, affected the growth, levels of oxidative stress markers (proline, malondialdehyde), and antioxidative enzymatic machinery in black gram grown in uncontaminated soil in pots and irrigated with solutions containing arsenic of above concentrations and in the presence or absence of Pi fertilization/application.

## 2. Materials and Methods

### 2.1. Plant Material and Arsenic Treatments

Black gram (*Vigna mungo* L. Hepper var. T9) seeds obtained from the authorized agency were surface sterilized with mercuric chloride (0.1%, w/v) and washed thoroughly and presoaked in petri dish for 4 hours containing sodium arsenate (Na_2_HAsO_4_·7H_2_O) solution of the following concentrations 0 *μ*M (control), 100 *μ*M, and 200 *μ*M with or without 40 ppm K_2_HPO_4_ solution. Seeds were then sowed in earthen pots having alluvial soil and compost (in the ratio 3 : 1). 500 mL of arsenic solution of above concentration was given weekly in each pot kept in wire house. Plants were analyzed for all parameters after 30 days of sowing except germination percentage which was evaluated after 5 days of sowing:

(1) Germination  %    =Number  of  germinated  seeds  in  a  pot  Total  number  of  seeds  sown  in  a  pot×100.

### 2.2. Morphological Studies

The root and shoot lengths together with their fresh weights of arsenic treated and untreated (control) black gram plants were measured after washing and rinsing plants with tap and deionised distilled water, respectively. Dry weight of roots and shoots was determined after oven drying at 70°C for 48 hours.

### 2.3. Chlorophyll and Carotenoid Contents

Total chlorophyll, chlorophyll a, and chlorophyll b contents were measured according to Arnon [29]. 100 mg leaves were crushed in 10 mL 80% chilled acetone. Extract was centrifuged at 2000 ×g for 10 minutes. Absorbance of supernatant was estimated spectrophotometrically at 645 nm and 663 nm using spectrophotometer (Toshniwal TSUV 75). Chlorophyll contents were expressed in terms of mg chlorophyll present/g fresh weight of tissue. Carotenoid contents were estimated according to method of Duxbury and Yentsch [30]. 

### 2.4. Malondialdehyde Contents (Lipid Peroxidation)

Lipid peroxidation in terms of malondialdehyde (MDA) was determined to access the membrane damage in black gram plants. For the measurement of lipid peroxidation, TBA (thiobarbituric acid) test was used to measure MDA level as an end product of lipid peroxidation [31]. 

### 2.5. Proline Contents

Proline contents estimated according to Bates et al. [32]. One g tissue of black gram plants was extracted with 5 mL of 0.1 M sulphosalicylic acid and centrifuged at 5000 ×g for 30 minutes. Two mL of supernatant, 5 mL glacial acetic acid, and 5 mL of 140 mM acid ninhydrin were added and shaken vigorously. The mixture was heated in a boiling water bath, and after cooling, the mixture was extracted in 10 mL of toluene in a separating funnel, and aqueous layer was discarded. The absorbance of the mixture was measured at 520 nm. The proline content was calculated from standard curve and expressed as *μ*M proline/100 mg fresh weight. 

### 2.6. Electrolyte Leakage Percentage (ELP)

ELP was calculated according to Sullivan and Ross [33]. Twenty leaf discs were incubated in distilled water at 25°C for 2 hour in test tubes, and initial conductivity (*E*1) of the bathing medium was measured. The tubes containing the discs were heated at 45°C to 55°C for 30 minutes to release electrolytes, cooled to 25°C, and conductivity was measured (*E*2). Later, the contents were boiled to 100°C for 10 minutes, and conductivity was measured after cooling to room temperature (*E*3). ELP was calculated as follows:

(2) $$\begin{array}{c} \text{ELP}=\frac{\left(E2-E1\right)}{E3}\times 100. \end{array}$$

### 2.7. Antioxidative Enzymes

Activity of catalase (CAT) enzyme was estimated according to Euler et al. [34]. Peroxidase (POX) activity was estimated according to Luck [35]. Superoxide dismutase (SOD) activity was assayed by the method of Beauchamp and Fridovich [36] by measuring its ability to inhibit the photochemical reduction of nitro blue tetrazoliun (NBT). Ascorbate peroxidase (APX) activity was determined by measuring the decrease in absorbance at 290 nm (*ɛ* = 2.8 mM^−1^ cm^−1^) due to oxidation of ascorbic acid to dehydroascorbate [37].

### 2.8. Protein

Protein contents were determined according to Lowry et al. [38] using bovine serum albumin as a calibration standard.

### 2.9. Statistical Analysis

The experiment was conducted in a completely randomized design (CRD) with 3 replications. The data were analyzed by One-way ANOVA using software program Sigmastats 3.5. It was followed by comparison of mean values using Holm Sidak method at *P* ≤ 0.05.

## 3. Results

### 3.1. Plant Growth, Biomass, Germination Percentage, and Soluble Protein Content

**Figure 1:**
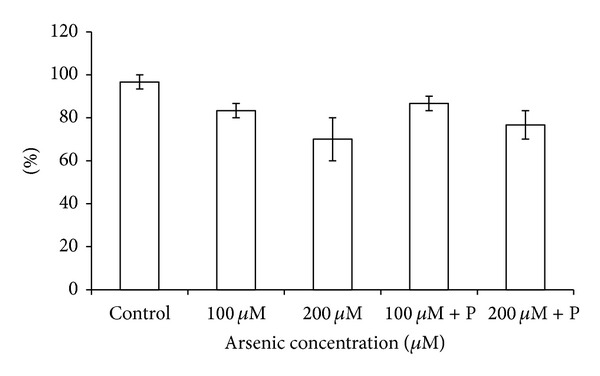
Effect of arsenate applied either singly or in combination with phosphate on the germination percentage of black gram. Error bars are the ± SE of three replicates; P = 40 ppm K_2_HPO_4_.

**Figure 2:**
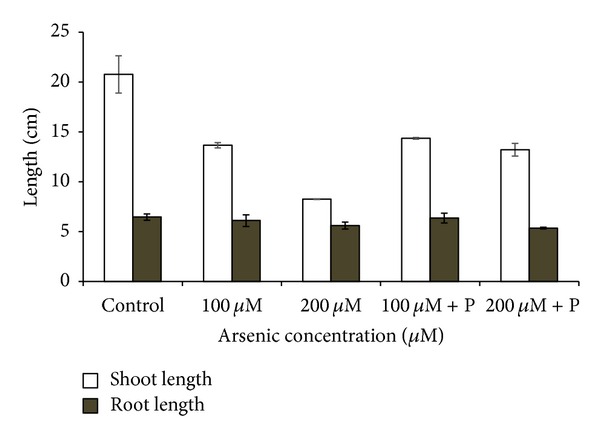
Effect of arsenate applied either singly or in combination with phosphate on the shoot and root length of black gram. Error bars are the ± SE of three replicates; P = 40 ppm K_2_HPO_4_.

**Figure 3:**
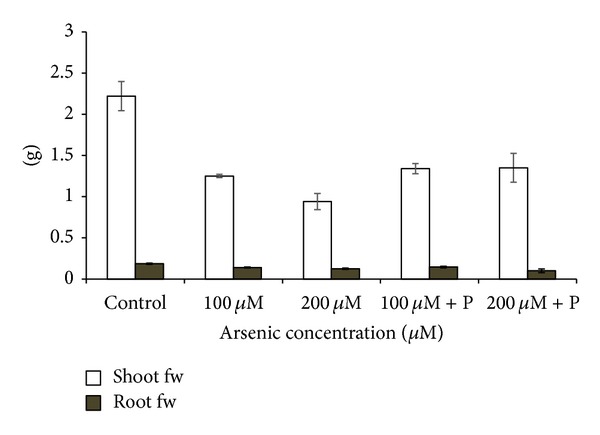
Effect of arsenate applied either singly or in combination with phosphate on the shoot and root fresh weight of black gram. Error bars are the ± SE of three replicates (fw = fresh weight); P = 40 ppm K_2_HPO_4_.

**Figure 4:**
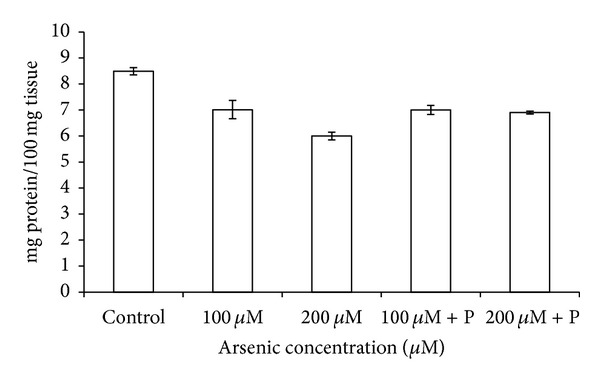
Effect of arsenate applied either singly or in combination with phosphate on the protein content of black gram. Error bars are the ± SE of three replicates; P = 40 ppm K_2_HPO_4_.

 Arsenic exposure significantly affected the normal growth and development of black gram plants. The root and shoot length of plants was decreased in higher arsenic doses (Figure 2). When black gram plants treated with arsenic along with phosphate were analyzed, we found that plant height and biomass increased as compared to only arsenic treatments (Table 1; Figures 2 and 3). Germination percentage decreased on increasing the applied arsenic concentrations. From 96.67%, in control, it declined to 70% in highest arsenic dose. However, on phosphate application, it increased to a substantial level (Figure 1). Soluble protein content also showed a decrease on arsenic application (Table 1; Figure 4). 

### 3.2. Photosynthetic Pigment Levels

**Figure 5:**
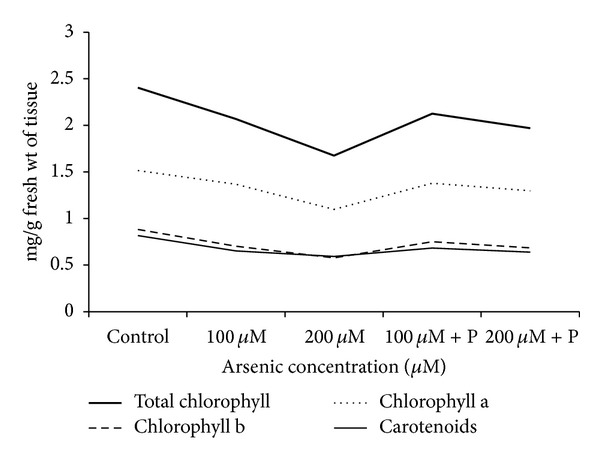
Effect of arsenate applied either singly or in combination with phosphate on the pigment contents. P = 40 ppm K_2_HPO_4_.

Total chlorophyll, chlorophyll a, b, and carotenoids all were decreased with increasing concentrations of arsenic accompanied by pale green coloration of leaves. In case of joint application of phosphate with arsenic, pigment contents were increased to a great amount (Table 2; Figure 5).

### 3.3. Oxidative Stress Markers

**Figure 6:**
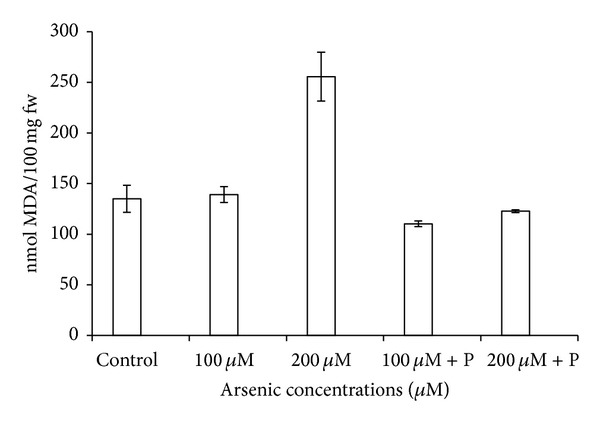
Effect of arsenate applied either singly or in combination with phosphate on the lipid peroxidation in black gram. Error bars are the ± SE of three replicates (MDA = malondialdehyde; fw = fresh weight); P = 40 ppm K_2_HPO_4_.

**Figure 7:**
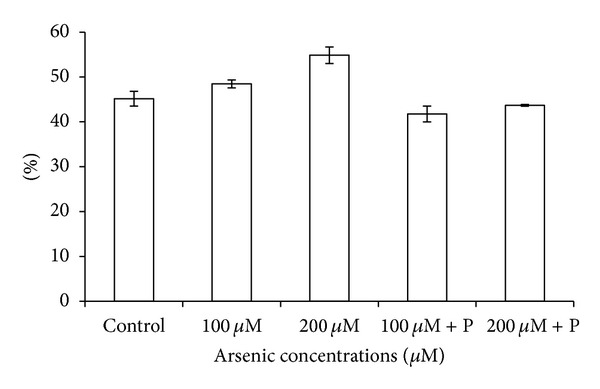
Effect of arsenate applied either singly or in combination with phosphate on electrolyte leakage percentage in black gram. Error bars are the ± SE of three replicates; P = 40 ppm K_2_HPO_4_.

**Figure 8:**
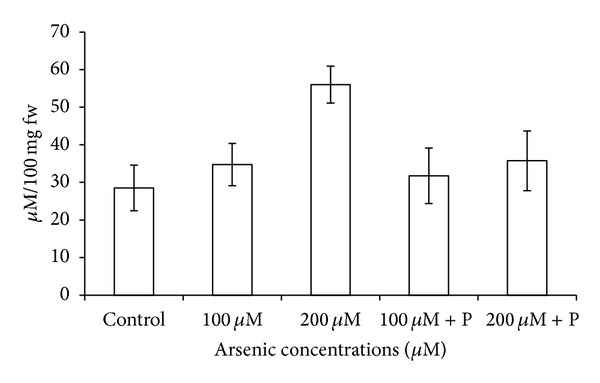
Effect of arsenate applied either singly or in combination with phosphate on the proline levels of black gram. Error bars are the ± SE of three replicates; P = 40 ppm K_2_HPO_4_.

Arsenic caused electrolyte leakage from leaves, and ELP was highest in 200 *μ*M arsenic treatment. However, joint application of phosphate did provide a marginal relief in the form of lowered ELP (Figure 7). Enhanced rate of lipid peroxidation was recorded as indicated by gradually increasing malondialdehyde (MDA) contents in plants exposed to arsenic. MDA contents increased to 52% in 200 *μ*M arsenic treatment (Figure 6). When phosphate was applied jointly with arsenic, elevated levels of MDA suffered considerable reduction. Proline content showed an increase on application of arsenic whereas application of phosphate together with arsenic significantly reduced proline content (Table 3; Figure 8). 

### 3.4. Antioxidative Enzymes

**Figure 9:**
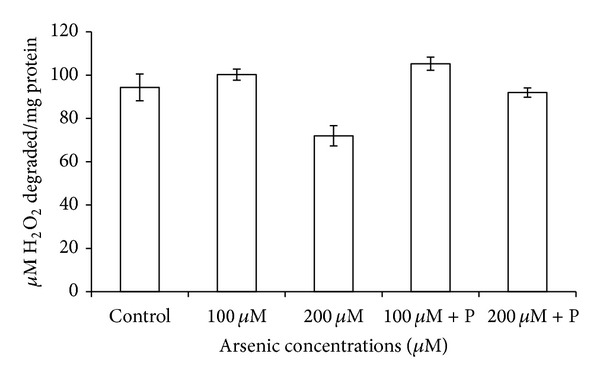
Effect of arsenate applied either singly or in combination with phosphate on the catalase activity of black gram. Error bars are the ± SE of three replicates; P = 40 ppm K_2_HPO_4_.

**Figure 10:**
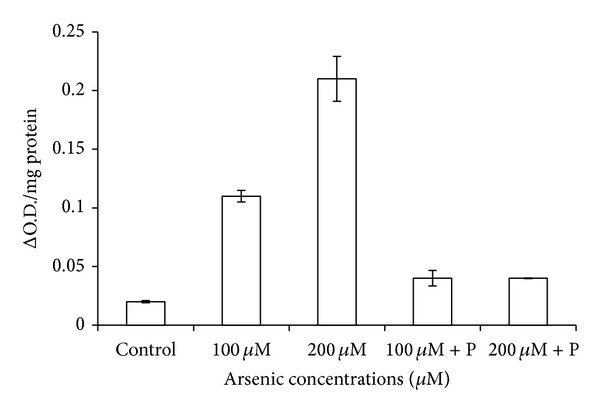
Effect of arsenate applied either singly or in combination with phosphate on the peroxidase activity of black gram. Error bars are the ± SE of three replicates; P = 40 ppm K_2_HPO_4_.

**Figure 11:**
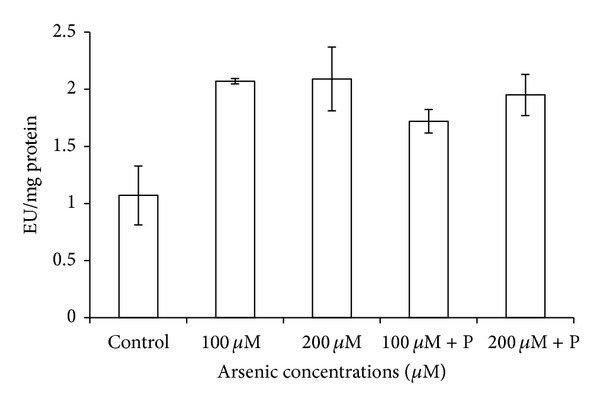
Effect of arsenate applied either singly or in combination with phosphate on the activity of superoxide dismutase in black gram. Error bars are the ± SE of three replicates; P = 40 ppm K_2_HPO_4_.

**Figure 12:**
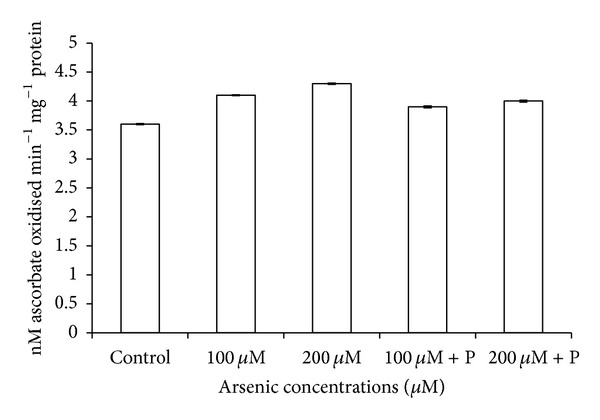
Effect of arsenate applied either singly or in combination with phosphate on the activity of ascorbate peroxidase in black gram. Error bars are the ± SE of three replicates; P = 40 ppm K_2_HPO_4_.

 The catalase activity was decreased in response to arsenic treatment of 200 *μ*M. On the contrary, a relief in the rate of reduction of CAT activity was observed in case of joint application of phosphate with arsenic (Table 3; Figure 9). CAT is an H_2_O_2_ splitting enzyme, and due to its low activity, level of H_2_O_2_ probably increased in plants which resulted in reduction in their growth. However, levels of other antioxidative enzymes like APX, POX, and SOD increased on arsenic exposure (Table 3; Figures 10, 11, and 12).

## 4. Discussion

The arsenic treatment resulted in symptoms of phytotoxicity and in considerable inhibition of initial growth of young black gram plants. There was chlorosis as well as necrosis of leaf tips. It was followed by senescence of leaves. Also, growth of plants was greatly hampered. It was observed that plant biomass together with height decreased markedly with increasing arsenic in treatment solution. The inhibitory effect of arsenic on root growth is in accordance with earlier reports in other plant species [39–41]. For plants grown without P addition (P−), there was a significant decrease in biomass production. However, it was less severe in case of As +P treatments. These findings are similar to results reported for rice [42, 43] and wheat [42, 44].

 The significant decrease of pigment contents in arsenic-treated plants is a sign of absence of adaptive adjustments of pigment synthesis to high arsenic levels. In previous works too, increased arsenic levels resulted in change of the chloroplast shape with concaving membrane bending and partial destruction together with the changes in the accumulation and flow of assimilates that leads to the decrease of chlorophyll contents in rice leaf [45].

 Increased lipid peroxidation in response to arsenic toxicity demonstrates increased generation of reactive oxygen species (ROS) in respect of oxidative stress. This is in accordance with previous reports that arsenic caused severe lipid peroxidation in mung bean [46], bean [47], and *Pteris* spp. [48, 49]. An enhancement in MDA levels denotes occurrence of membrane damage resulting from peroxidation of polyunsaturated fatty acids, which causes generation of ROS and ensuing oxidative stress [50]. Arsenic induced membrane damage was also apparent from an increased electrolyte leakage (EL) from the leaves. ELP is an indicator of membrane damage and primarily occurs due to peroxidation of membrane that results from an oxidative burst [51]. Proline acts as a cytoplasmic osmoticum and also protects the protein against denaturation [52]. Proline content, here, acting as stress marker for oxidative damage increased on application of arsenic whereas application of phosphate together with arsenic induced significant reduction of proline content.

 It is well known that heavy metal toxicity of plants results into complex biochemical responses and many defensive mechanisms meant to detoxify ROS that readily occur in plants due to metal contamination [53, 54]. APX, CAT, SOD, POX are regarded as key enzymes within the antioxidative defense mechanism, which directly determines the cellular concentration of oxygen radicals, and H_2_O_2_. SOD is considered as the major superoxide (O_2_^•−^) scavenger and also provides first line of defense against cellular injury due to environmental stress [55]. The highly reactive O_2_^−^ is converted to H_2_O_2_ by SOD [56]. CAT is a heme containing tetrameric protein and one of the well-known H_2_O_2_ splitting enzymes [56]. Excess H_2_O_2_ generated due to SOD activity is detoxified by CAT. However, here, a significant decline in CAT activity at higher arsenic dose suggests that this enzyme is not involved in protection of black gram plants against arsenic toxicity. On the other hand, increased activity of POX and SOD enzymes on arsenic application suggests their protective role in arsenic toxicity. Results show an increase in the activity of APX in response to arsenic stress. APX utilizes the reducing power of ascorbic acid to remove potentially harmful H_2_O_2_ [48].

 Arsenate acts as a Pi analogue and is transported across the plasma membrane via a Pi cotransport systems [26]. Once it is inside the cytoplasm, arsenate competes with Pi, for example, replacing Pi in ATP to form unstable ADP-As. This leads to the disruption of energy flows in cells [42]. Also, in arsenate resistant plants with high P status a reduced As sensitivity has been noticed, which is not due to a difference in arsenate influx, but is probably a result of higher cytoplasmic Pi status, which decreases arsenate toxicity within the cell [53]. The effects due to P nutrition on metabolism of arsenate could be the following: (1) high plant P status that leads to a down regulation of the arsenate/Pi plasma lemma transporters; (2) high cellular Pi levels results in greater competition with arsenate for biochemical processes where arsenate substitutes for Pi [57]. Here also, all the parameters showed positive alterations in the presence of phosphate leading to better growth and metabolism in black gram. Arsenate tolerance could be enhanced by increasing Pi uptake [42]. Also, P nutrition could be involved in the reduction of reactive oxygen species and non protein thiols production, formed during exposure to As that cause tissue damage and lipid peroxidation [41]. P fertilization may reduce the impact of arsenic toxicity without increase in arsenic concentrations in above ground parts of plants [42]. 

## 5. Conclusion

The detrimental effect of toxic arsenic in black gram was clearly evident in the form of retarded growth and reduced biomass. This was further supported by elevated activity of oxidative stress markers and anti oxidative enzymes. This clearly shows that black gram is quite sensitive towards arsenic toxicity. However, phosphate application ameliorated to a large extent, the damaging effects caused by arsenic toxicity. This has practical importance in agricultural systems, as can reduce yield losses and also improve quality of crops.

## Figures and Tables

**Table 1:** Effect of arsenate applied either singly or in combination with phosphate on the height, biomass, germination % and protein content of black gram.

	Control	100 *µ*M As	200 *µ*M As	100 *µ*M As + P	200 *µ*M As + P
Germination %	96.67 ± 3.330	83.33 ± 3.330	70.00 ± 10.000	86.66 ± 3.330	76.66 ± 6.670
Shoot length (cm)	20.75 ± 1.876	13.65 ± 0.260*	8.25 ± 0.029*	14.35 ± 0.087*	13.20 ± 0.635*
Root length (cm)	6.45 ± 0.318	6.10 ± 0.577	5.60 ± 0.346	6.35 ± 0.491	5.35 ± 0.087
Shoot fresh weight (g)	2.22 ± 0.176	1.25 ± 0.020*	0.94 ± 0.098*	1.34 ± 0.061*	1.35 ± 0.176*
Shoot dry weight (g)	0.35 ± 0.032	0.17 ± 0.003*	0.15 ± 0.000*	0.22 ± 0.003*	0.19 ± 0.017*
Root fresh weight (g)	0.18 ± 0.009	0.14 ± 0.006	0.12 ± 0.009*	0.14 ± 0.009*	0.10 ± 0.023*
Root dry weight (g)	0.027 ± 0.001	0.016 ± 0.000	0.014 ± 0.002	0.016 ± 0.001	0.013 ± 0.001
Protein (mg/100 mg tissue)	8.49 ± 0.141	7.01 ± 0.352*	6.00 ± 0.147*	7.00 ± 0.173*	6.90 ± 0.058*

P = 40 ppm K_2_HPO_4_; values are means of three replicates ± SE *Data significant at *P* < 0.05. Multiple comparisons versus control group (Holm Sidak method) overall significance level = 0.05.

**Table 2:** Effect of arsenate applied either singly or in combination with phosphate on the pigment levels in black gram.

Treatments	Total chlorophyll (mg/g fw of tissue)	Chlorophyll a (mg/g fw of tissue)	Chlorophyll b (mg/g fw of tissue)	Carotenoids (mg/g fw of tissue)
Control	2.40 ± 0.006	1.51 ± 0.003	0.88 ± 0.009	0.82 ± 0.002
100 *µ*M As	2.07 ± 0.007*	1.37 ± 0.001*	0.70 ± 0.006*	0.65 ± 0.002*
200 *µ*M As	1.67 ± 0.004*	1.10 ± 0.002*	0.58 ± 0.002*	0.59 ± 0.002*
100 *µ*M As + P	2.13 ± 0.010*	1.38 ± 0.005*	0.75 ± 0.005*	0.68 ± 0.006*
200 *µ*M As + P	1.97 ± 0.005*	1.30 ± 0.008*	0.69 ± 0.004*	0.64 ± 0.005*

P = 40 ppm K_2_HPO_4_; values are means of three replicates ± SE *Data significant at *P* < 0.05. Multiple comparisons versus control group (Holm Sidak method) overall significance level = 0.05.

**Table 3:** Effect of arsenate applied either singly or in combination with phosphate on the oxidative stress elements and anti oxidative enzymes in black gram.

	Control	100 *µ*M As	200 *µ*M As	100 *µ*M As + P	200 *µ*M As + P
Lipid peroxidation (m mol MDA/100 mg fw)	135.00 ± 13.330	139.19 ± 7.870	255.60 ± 24.160*	110.36 ± 2.860	122.76 ± 1.430
ELP (%)	45.15 ± 1.640	48.44 ± 0.900	54.83 ± 1.860*	41.75 ± 1.760	43.65 ± 0.200
Proline (*µ*M/100 mg fw)	28.50 ± 6.060	34.75 ± 5.630	56.00 ± 4.910	31.75 ± 7.360	35.75 ± 7.940
Catalase (*µ*M H_2_O_2 _degraded/mg protein)	94.31 ± 6.230	100.24 ± 2.560	71.95 ± 4.650*	105.22 ± 3.020	91.97 ± 2.160
Peroxidase (ΔO.D./mg protein)	0.02 ± 0.001	0.11 ± 0.005*	0.21 ± 0.019*	0.04 ± 0.006	0.04 ± 0.003
SOD (EU/mg protein)	1.07 ± 0.257	2.07 ± 0.023*	2.09 ± 0.279*	1.72 ± 0.103*	1.95 ± 0.180*
APX (nM ascorbate oxidized min^−1^ mg^−1 ^protein).	3.60 ± 0.011	4.10 ± 0.006*	4.30 ± 0. 011*	3.90 ± 0. 017*	4.00 ± 0.017*

P = 40 ppm K_2_HPO_4_; values are means of three replicates ± SE *Data significant at *P* < 0.05. Multiple comparisons versus control group (Holm Sidak method) overall significance level = 0.05.
